# Retraction: lncRNA ABHD11‐AS1, regulated by the EGFR pathway, contributes to the ovarian cancer tumorigenesis by epigenetically suppressing TIMP2

**DOI:** 10.1002/cam4.7098

**Published:** 2024-03-12

## Abstract

Xiang‐Yang Zeng, Xiao‐Yan Jiang, Jia‐Hui Yong, Hui Xie, Jing Yuan, Da Zeng, Ying‐Yu Dou, Song‐Shu Xiao “lncRNA ABHD11‐AS1, regulated by the EGFR pathway, contributes to the ovarian cancer tumorigenesis by epigenetically suppressing TIMP2” Cancer Med. 2019; 8: 7074–7085 (https://doi.org/10.1002/cam4.2586)

The above article, published online on 30 September 2019, in Wiley Online Library (wileyonlinelibrary.com), has been retracted by agreement between the journal's Editor‐in‐Chief, Stephen Tait, and John Wiley and Sons Ltd. Following publication, concerns were raised by a third party regarding image overlap within Figure 2b. Furthermore, several figure elements have been published elsewhere, identifying the cells as originating from different cell lines and subjected to different treatment. The authors did not respond to the concerns raised and did not provide the original data. The editors consider the results and conclusion reported in this article unreliable.

Xiang‐Yang Zeng, Xiao‐Yan Jiang, Jia‐Hui Yong, Hui Xie, Jing Yuan, Da Zeng, Ying‐Yu Dou, Song‐Shu Xiao “lncRNA ABHD11‐AS1, regulated by the EGFR pathway, contributes to the ovarian cancer tumorigenesis by epigenetically suppressing TIMP2” Cancer Med. 2019; 8: 7074–7085 (https://doi.org/10.1002/cam4.2586)

The above article, published online on 30 September 2019, in Wiley Online Library (wileyonlinelibrary.com), has been retracted by agreement between the journal's Editor‐in‐Chief, Stephen Tait, and John Wiley and Sons Ltd. Following publication, concerns were raised by a third party regarding image overlap within Figure 2b. Furthermore, several figure elements have been published elsewhere, identifying the cells as originating from different cell lines and subjected to different treatment. The authors did not respond to the concerns raised and did not provide the original data. The editors consider the results and conclusion reported in this article unreliable.

